# An expanded clinical spectrum of hypoinsulinaemic hypoketotic hypoglycaemia

**DOI:** 10.1186/s13023-023-02954-5

**Published:** 2023-11-16

**Authors:** Alena Welters, Sarah M Leiter, Nadine Bachmann, Carsten Bergmann, Henrike Hoermann, Eckhard Korsch, Thomas Meissner, Felicity Payne, Rachel Williams, Khalid Hussain, Robert K. Semple, Sebastian Kummer

**Affiliations:** 1 Department of General Paediatrics, Neonatology and Paediatric Cardiology, Medical Faculty, University Children’s Hospital, Heinrich-Heine University, Düsseldorf, Germany; 2 MRC Metabolic Diseases Unit, Wellcome-MRC Institute of Metabolic Science, University of Cambridge, Cambridge, UK; 3 Medizinische Genetik Mainz, Limbach Genetics, Mainz, Germany; 4 Paediatric Endocrinology, Children’s Hospital, Amsterdamer Straße 59, Cologne, Germany; 5 Department of Paediatrics, University of Cambridge, Cambridge, UK; 6 Department of Paediatric Medicine, Division of Endocrinology and Diabetes, Sidra Medicine, Education City North Campus, Doha, Qatar; 7 Centre for Cardiovascular Science, The University of Edinburgh, Edinburgh, UK; 8 MRC Human Genetics Unit, Institute of Genetics and Cancer, The University of Edinburgh, Edinburgh, UK

**Keywords:** Hypoinsulinemic hypoglycaemia, PI3K, Pseudohyperinsulinism, Insulin signalling

## Abstract

**Background:**

Hypoketotic hypoglycaemia with suppressed plasma fatty acids and detectable insulin suggests congenital hyperinsulinism (CHI). Severe hypoketotic hypoglycaemia mimicking hyperinsulinism but without detectable insulin has recently been described in syndromic individuals with mosaic genetic activation of post-receptor insulin signalling. We set out to expand understanding of this entity focusing on metabolic phenotypes.

**Methods:**

Metabolic profiling, candidate gene and exome sequencing were performed in six infants with hypoketotic, hypoinsulinaemic hypoglycaemia, with or without syndromic features. Additional signalling studies were carried out in dermal fibroblasts from two individuals.

**Results:**

Two infants had no syndromic features. One was mistakenly diagnosed with CHI. One had mild features of megalencephaly-capillary malformation-polymicrogyria (MCAP) syndrome, one had non-specific macrosomia, and two had complex syndromes. All required intensive treatment to maintain euglycaemia, with CHI-directed therapies being ineffective. Pathogenic *PIK3CA* variants were found in two individuals – *de novo* germline c.323G>A (p.Arg108His) in one non-syndromic infant and postzygotic mosaic c.2740G>A (p.Gly914Arg) in the infant with MCAP. No causal variants were proven in the other individuals despite extensive investigation, although rare variants in mTORC components were identified in one. No increased PI3K signalling in fibroblasts of two individuals was seen.

**Conclusions:**

We expand the spectrum of PI3K-related hypoinsulinaemic hypoketotic hypoglycaemia. We demonstrate that pathogenic germline variants activating post-insulin-receptor signalling may cause non-syndromic hypoinsulinaemic hypoketotic hypoglycaemia closely resembling CHI. This distinct biochemical footprint should be sought and differentiated from CHI in infantile hypoglycaemia. To facilitate adoption of this differential diagnosis, we propose the term “pseudohyperinsulinism”.

**Supplementary Information:**

The online version contains supplementary material available at 10.1186/s13023-023-02954-5.

## Background

Hypoglycaemia is the commonest neonatal metabolic emergency, and has diverse causes including hyperinsulinism, hypopituitarism, and disorders of glycogen storage or fatty acid oxidation. Hypoketotic hypoglycaemia with suppressed plasma fatty acids and detectable insulin suggests congenital hyperinsulinism (CHI), the most frequent cause of persistent neonatal hypoglycaemia [1].

Hypoketotic hypoglycaemia may also be seen without detectable insulin in fatty acid oxidation disorders. Recently, this has additionally been attributed in some children to genetic activation of the phosphoinositide 3-kinase (PI3K)–AKT-mTOR signalling cascade, a crucial component of intracellular insulin signalling [2–5]. Pathogenic variants in the cascade lead to autonomous activation of “insulin signalling” in target tissues, inducing severe hypoglycaemia without detectable insulin. PI3K–AKT-mTOR signalling also plays a critical role in the growth-promoting effects of insulin, IGF1 and other growth factors. Thus, different degrees of asymmetric or segmental overgrowth have been a hallmark of this group of hypoglycaemic disorders to date [6].

Individuals with the activating c.49G>A (p.Glu17Lys) variant in *AKT2* exhibit severe hypoketotic hypoinsulinaemic hypoglycaemia from the first few months of life with mild to moderate somatic hemihypertrophy [3, 7–9]. Activating mutations in *PIK3CA*, encoding a critical PI3K catalytic subunit, produce a similar biochemical profile of hypoketotic hypoinsulinaemic hypoglycaemia, associated with syndromic overgrowth falling in the *PIK3CA*-related overgrowth spectrum (PROS) [4, 10]. This is particularly seen in the megalencephaly-capillary malformation-polymicrogyria (MCAP) syndrome (OMIM: 602501) [11, 12]. Hypoketotic hypoinsulinaemic hypoglycaemia has also been associated with brain overgrowth caused by mutations in *PIK3R2*, encoding a PI3K regulatory subunit [4, 10]. However, in most reported individuals somatic overgrowth rather than the metabolic profile was the sentinel clinical feature pointing towards dysregulation of the PI3K-AKT-mTOR pathway. It has thus been suggested that individuals with segmental overgrowth should routinely be screened for hypoglycaemia [13].

We now describe six further infants with severe hypoketotic, hypoinsulinaemic hypoglycaemia, among whom only four had associated syndromic features. In two infants, including one non-syndromic individual, pathogenic *PIK3CA* mutations were found. Our report illuminates the variability of *PIK3CA*-related hypoglycaemic disorders, and extends the spectrum of congenital hypoglycaemia.

## Materials and methods

Six individuals (individual 1–6; I1-6) with severe hypoketotic hypoglycaemia were studied. I1-3 were investigated as part of clinical care and their guardians gave written informed consent to be included in this report. I4-6 were studied as part of a genetic research study approved by the UK Research Ethics Committee, with full informed consent.

### Clinical evaluation

Metabolic profiling included measurement of insulin and C-peptide, free fatty acids and betahydroxybutyrate during hypoglycaemia of ≤ 3 mmol/L. Biochemical analyses were performed in accredited laboratories. Initial glucose requirement is defined as the amount that was used for therapeutic management. In some individuals, minimum carbohydrate requirement was assessed by formal titration of continuous i.v. glucose infusion to maintain euglycaemia (3.9 mmol/L and 5 mmol/L, only performed for I2 and I4).

### Genetic studies

Genetic investigations included next generation sequencing (NGS) of gene panels, exomes and/or targeted sequencing of overgrowth-related genes including *AKT2* and *PIK3CA*, as specified for each individual studied. Exome sequencing for I4-6 was performed essentially as previously described for the UK10K Project [14]. If no pathogenic or likely pathogenic variant was identified by targeted sequencing, exome sequencing of the proband and unaffected parents was undertaken, with trio analysis focusing on *de novo*, homozygous or compound heterozygous variants altering coding sequence.

Those unlikely to have a functional impact based on bioinformatic prediction tools and all variants with a non-reference allele frequency greater than 1 in 100,000 in the GnomAD repository ([15]; accessed July 2021) were excluded. Specific analysis for mosaic mutations was performed within exome datasets (I1) or using a custom next generation sequencing panel (I4-6). Non-synonymous, exonic variants with a read depth > 50, and quality > 10 were extracted.

### Cellular studies

Dermal fibroblasts were isolated from I4 and I5. These were cultured and analysed for basal phosphorylation of AKT1/2 (Thr308/309 and Ser473/474) and GSKβ (Ser9) as previously described [4].

## Results

### Clinical case histories

Clinical characteristics and treatment of all individuals are summarized in Table 1. We categorised them as having (I) non-syndromic, (II) mildly syndromic, (III) overgrowth-dominated or syndromic pseudohyperinsulinism.

**Table 1:** Clinical characteristics, metabolic profile and treatment of individuals studied

		Individual 1					Individual 2						Individual 3
**Gestational age (w); birth weight (g), head circumference (cm)**		37+0; 3430 (+ 0.74 SD), 37.5 (+ 2.07 SD)					37+2; 2215 (-2.14 SD), 33.5 (-0.71 SD)						34+4; 3470 (+ 2.25SD), 36.5 (+ 2.3 SD)
**Sex**		male					male						male
**Typical PI3K-associated features**		haemangioma on third digit of left hand					none						macrocephaly, hydrocephalus with low-lying cerebellar tonsils requiring VP-shunt, cutis marmorata, telangiectasia (face/neck), mild secondary hypothyroidism
**Other clinical features**		left-sided neonatal stroke with secondary haemorrhage (associated with a lipoprotein (Lp)a glycoprotein variant), small VSD and ASD, infantile esotropia, hyperopia, astigmatism					umbilical hernia, transient neonatal cholestasis: max. direct bilirubin 4.72 mg/dl (ref < 1 mg/dl), gGT max. 455 U/l (ref < 200 U/l), AP max. 892 U/l (ref < 469 U/l)						glandular hypospadia, undescended testes, transient total hyperbilirubinaemia, marginally low cortisol response to CRH stimulation
**Developmental delay**		yes, mild (presumably associated with neonatal stroke)					no						yes, mild
**Age at genetic diagnosis (y)**		12					N/A						1.25
**Age at diagnosis of hypoglycaemia**		day 1 of life					day 1 of life						2 months
**Metabolic profile during hypoglycaemia**	**Day of life/age**	**11**	**17**	**35**	**38**	**39**	**6**	**21**		**39**	**45**		**2 month old**
	Glucose (mmol/L)	2.3	1.9	2.3	1.7	0.8	2.4	2.3		2.3	3		2.9
	Insulin (mU/L)	3.9	0.6	< 0.1	0.8	0.5	0.8	0.6		0.7	0.02		1.3
	C-peptide (ng/ml)	1.66	0.1	n.d.	n.d.	0.3	0.31	n.d.		0.67	0.07		1.38
	FFA (mmol/L)	0.34	0.34	0.01	0.15	0.15	0.37	< 0.1		n.d	n.d		0.22
	BHB (mmol/L)	0.51	0.01	0.23	0.01	0.01	n.d	< 0.1		n.d	0.4		0.2
**Pathogenic mutation**		*de novo* germline mutation in *PIK3CA* (c.323G>A p.(Arg108His))					none detected						*de novo* mosaic mutation in *PIK3CA* (c.2740G>A p.(Gly914Arg))
**Additional investigations related to hypoglycaemia**		^18^F-DOPA PET/CT: diffuse tracer uptake; biochemically no indication of free fatty acid oxidation disorders; organic acid disorders; urea cycle disorders; congenital disorders of glycosylation; no mutation in known CHI genes†					no mutation in known CHI genes†						no mutation in known CHI genes†
**Carbohydrate amounts initially required for therapeutic management (without formal titration)**		16 mg/kg/min at day 12 of life (i.v. and oral)					6.4 mg/kg/min at day 3 of life (i.v. and oral)						10 mg/kg/min at 2 months of age
**Formal testing of minimum glucose requirement (titrated continuous i.v. glucose infusion)**		not performed					4 mg/kg/min at 6 weeks of life						not performed
**Medication for hypoglycaemia (initial dosage)**		unresponsive to DZX (15 mg/kg/d), improvement on somatostatin infusions (15 µg/kg/d) in parallel with nutritional change					unresponsive to DZX (15 mg/kg/d)						unresponsive to hydrocortisone (20 mg/m^2^)
**HH medication-related side effects**		DZX: oedema, hypertrichosis; somatostatin: gall bladder sludge					none						none
**PEG feeding**		no					no						no
**Feeding regime at discharge (after diagnosis of hypoglycaemia)**		at 2 months of age: starch-enriched meals every 6 h					at 2 months of age: starch-enriched meals every 3–4 h						at 2 months of age: starch-enriched meals every 4 h
**Age at patient’s last clinic visit**		12 years					4 months						1 year
**Current management of hypoglycaemia**		at 12 years of age: none; normal fasting tolerance, at least 15 h (not formally assessed)					at 3 years of age: none; fasting tolerance not formally assessed						at 12 months of age: none; fasting tolerance 12 h
		**Individual 4**					**Individual 5**						**Individual 6**
**Gestational age (w); birth weight (g); head circumference (cm)**		41; 3120 (-1.07 SD); 34 (+ 0.1 SD)					35; 3050 (+ 1.22 SD); 35 (+ 1.10 SD)						36+1; 3150 (+ 0.57 SD)
**Sex**		female					female						male
**Typical PI3K-associated features**		none					left sided hemi-hypertrophy						right sided hemi-hypertrophy
**Other clinical features**		hypoxic ischaemic encephalopathy, meconium aspiration syndrome with neonatal sepsis, atrial septal defect					low set eyes, small mouth, small facial bones in comparison to the cranium, round and small labia majora, bilateral cystic nephropathy, high grade adrenal tumour						multiple jejunal atresia
**Developmental delay**		yes, severe					none						none
**Age at genetic diagnosis (y)**		N/A					N/A						N/A
**Age at diagnosis of hypoglycaemia**		10 months					day 2 of life						day 1 of life
**Metabolic profile during hypoglycaemia**	**Day of life/age**	**13 months**					**2 months**		**18 months**			**24 months**	**6 weeks**
	Glucose (mmol/L)	2.4					1.3		1.1			1.7	2
	Insulin (mU/L)	< 5					< 1.5		< 1.5			< 1.5	< 0.3
	C-peptide (ng/ml)	< 0.3					ND		ND			ND	ND
	FFA (mmol/L)	0.35					0.37		ND			ND	0.6
	BHB (mmol/L)	0.05					0		ND			0	< 0.1
**Pathogenic mutation**		none detected					none detected						not proven
**Additional investigations related to hypoglycaemia**		mosaicism for *AKT2* c.49G>A excluded by RFLP; no basal AKT or GSK3 hyperphosphorylation in dermal fibroblasts; no mutations identified on high depth sequencing of overgrowth-associated genes					liver biopsy: normal; mosaicism for *AKT2 *c.49G>A excluded by RFLP; no basal AKT or GSK3 hyperphosphorylation in dermal fibroblasts; no mutations identified on high depth sequencing of overgrowth-associated genes; no epigenetic or copy number abnormalities at chromosome 11p15 detected by methylation-sensitive MLPA						mosaicism for *AKT2* c.49G>A excluded by RFLP; no epigenetic or copy number abnormalities at chromosome 11p15 detected by methylation-sensitive MLPA
**Initial glucose requirement**		6 mg/kg/min					10–19 mg/kg/min in neonatal period						ND
**Formal testing of minimum glucose requirement (continuous i.v. glc infusion)**		6 mg/kg/min (13 months)					not performed						not performed
**Medication for hypoglycaemia (initial dosage)**		no response to DZX or sirolimus; prednisolone at 1 mg/kg					unresponsive to DZX						none
**HH medication-related side effects**		rapid weight gain in response to steroids					none						N/A
**PEG feedings**		yes					yes						no
**Feeding regime at discharge (after diagnosis of hypoglycaemia)**		regular bolus feeds during the day, overnight continuous PEG feed					four-hourly bolus feeds						initially parenteral nutrition due to short gut, then regular bolus feeds.
**Age at patient’s last clinic visit**		N/A – patient deceased					14 years						8 months
**Current management of hypoglycaemia**		N/A					frequent daytime meals and waking once at night to consume fruit juice enriched with starch and glucose.						fasting tolerance > 8 h

Abbreviations: AP, alkaline phosphatase; ASD, atrial septal defect; BHB, betahydroxybutyrate; CHI, congenital hyperinsulinism; DZX, diazoxide; FF, free fatty acids; HH, hyperinsulinaemic hypoglycaemia; MLPA, multiple ligation probe amplification; PEG, percutaneous endoscopic gastrostomy; VP-shunt, ventriculo-peritoneal shunt; VSD, ventricular septal defect; N/A, not applicable; ND, not determined

### Non-syndromic pseudohyperinsulinism

Individuals 1 and 2 (I1 and I2) were born at term to non-consanguineous parents of German and Guinean origin, respectively. Neither showed generalised or localised/asymmetric somatic overgrowth, although I1 had macrocephaly (OFC + 2.07 SD) at birth, which was not sustained (subsequent OFC < 2 SD). I2 was a dichorionic diamniotic twin born small for gestational age. Additional clinical features are listed in Table 1. In both individuals, severe hypoglycaemia (0.8 mmol/L and 1.1 mmol/L respectively) was noted on day one of life. Repeated metabolic assessments during hypoglycaemia revealed hypoketotic hypoglycaemia (Table 1) leading to suspicion of CHI. In I1 detectable plasma insulin was documented only once at 11 days of age (3.9 mU/L) at hypoglycaemia of 2.3 mmol/L. All other critical samples in hypoglycaemia in both individuals revealed appropriate suppression of plasma insulin levels ≤ 0.9 mU/L during hypoglycaemia, with ketones and free fatty acids suppressed (Table 1). CHI was still assumed the most likely diagnosis for I1. ^18^ F-L-Dopa-PET-CT showed homogeneous tracer uptake.

Treatment attempts used to maintain physiological blood glucose are summarised in Table 1. In both individuals diazoxide titrated to 15 mg/kg/d was ineffective. I1 was treated with subcutaneous octreotide infusion (15 µg/kg/d) from two weeks of age and starch-enriched meals every 3–4 h, but no clear response to octreotide distinct from the effect of nutrition could be discerned. Octreotide infusion was gradually reduced while fasting tolerance increased to 12 h at 12 months, and it was finally stopped at 20 months of age due to decreasing growth velocity. At this time, I1 could fast for at least 6 h on age-appropriate diet without hypoglycaemia (no formal extended fasting tolerance test performed). At 12 years’ old fasting of > 15 h is tolerated without hypoglycaemia.

I2 initially received starch-enriched feedings every 2 h during daytime, and nocturnal continuous feeding via nasogastric tube to prevent hypoglycaemia. At 2 months of age fasting tolerance had increased to 3–4 h on starch-enriched bolus feedings (equivalent to 13–17 mg/kg/min carbohydrates), and weaning from nocturnal tube feeding was achieved. However, formal assessment of glucose requirement by i.v. glucose titration revealed a requirement of only 4 mg/kg/min to maintain euglycaemia. Thus, oral bolus carbohydrate amount required to cover a certain fasting period may exceed carbohydrate needs during continuous i.v. glucose. At 4 months of age, euglycaemia was maintained on an oral starch-enriched feeding regimen equivalent to 9–11 mg/kg/min of carbohydrates. Fasting tolerance had increased to 5 h. Subsequently, even without specific treatment no clinical signs of hypoglycaemia were reported.

### Mildly syndromic pseudohyperinsulinism

Individuals 3 and 4 (I3 and I4) were born to healthy non-consanguineous parents, of Russian and Portuguese origin, respectively. Individual 3 (I3) was born large for gestational age at 34 + 4 weeks of gestation, with macrocephaly, glandular hypospadia and cryptorchidism noted at birth. Further mild MCAP-associated clinical features were noticed during clinical workup (Table 1). Individual 4 (I4) was delivered at term by Caesarean section due to foetal distress and intubated and ventilated for 12 h. She remained in intensive care for 25 days due to grade 1 hypoxic encephalopathy (according to Sarnat staging), meconium aspiration, and sepsis. No localized somatic overgrowth or hemihypertrophy were observed.

I3 did not show overt hypoglycaemic symptoms. Instead, mild syndromic features of MCAP syndrome prompted serial screening for hypoglycaemia at 2 months old, which repeatedly revealed hypoinsulinaemic hypoketotic hypoglycaemia of < 2.8–3.3 mmol/L upon 3–4 h of fasting. (Table 1). At 4 months of age, acute gastroenteritis led to hypoglycaemia < 2 mmol/L requiring intravenous glucose infusion.

I4 developed tonic-clonic seizures at 7 months’ old. At 10 months old increasing seizure frequency led to admission and severe spontaneous hypoglycaemia was noted. This was initially managed with 2-hourly nasogastric tube feeding, but at 13 months hypoglycaemia worsened. At this stage dysmorphic features (low set ears, short neck, short palpebral fissures) were observed, with mild hepatomegaly, severe obesity, obstructive hypoventilation requiring overnight respiratory support, and global developmental delay. Controlled fasting revealed hypoketotic hypoglycaemia without detectable insulin or C-peptide (Table 1). It was not before 5 years of age that subtle left-sided hemihypertrophy was noted.

Carbohydrate requirements and feeding regimens are summarized in Table 1. In I3 euglycaemia was achieved using starch-enriched meals every 4 h. At 2 months of age, oral carbohydrate requirement to maintain euglycaemia was equivalent to 10 mg/kg/min (no formal titration to minimum on continuous i.v. glucose). At 8 months of age nocturnal feeding with starch-enriched meals every 6 h was sufficient to maintain blood glucose > 3.3 mmol/L. At 12 months of age, fasting tolerance had increased to 12 h on an age-appropriate diet without additional carbohydrates.

For I4 glucose requirement to maintain euglycaemia was 6 mg/kg/min at 13 months old. No response of hypoglycaemia to diazoxide nor sirolimus, maintained at plasma levels of 2 ng/ml (2.2 nM), was seen. Prednisolone at 1 mg/kg/day was used to mitigate hypoglycaemia together with regular bolus feeds and overnight continuous feeding via percutaneous endoscopic gastrostomy (PEG). Between 13 months and 4.4 years’ old prednisolone was weaned, however idiopathic thrombotic thrombocytopenic purpura (TTP) was diagnosed and prednisolone increased to 40 mg/day. Over ensuing months weight gain and myopathy with obstructive sleep apnoea required insertion of a tracheostomy and ICU admission.

At 5 years old, on weaning of steroids, hypoglycaemia recurred, requiring 10% intravenous glucose, subcutaneous glucagon infusion, and continuous percutaneous feeding including 12 h of overnight feeding and 3-hourly starch-enriched boluses during the day. Fasting evaluation again confirmed hypoketotic hypoglycaemia with undetectable insulin and C-peptide as well as low free fatty acids. She was later reported to have died. Details of her terminal illness are not available.

#### Overgrowth-dominated or syndromic pseudohyperinsulinism

Individual 5 (I5) was born to non-consanguineous British parents by Caesarean section at 35 weeks due to polyhydramnios and premature rupture of membranes. Bilateral cystic nephrophathy (Potter class III) had been noted *in utero*. She weighed 3.05 kg (+ 1.22 SD), with length 48 cm (+ 0.05 SD), and head circumference 35 cm (+ 1.10 SD) at birth (Table 1). A protuberant abdomen and an epigastric mass, low set eyes, small mouth, small facial bones in comparison to the cranium, and small labia majora were noted. A large, heterogeneous left adrenal mass was observed by ultrasonography neonatally.

At 2 days of age hypoglycaemia (blood glucose nadir 0.4 mmol/L) required enteral and parenteral glucose averaging 10–19 mg/kg/day to maintain euglycaemia (no formal titration to minimum on continuous i.v. glucose). A fasting test at 2 months provoked hypoketotic, hypoinsulinaemic hypoglycaemia with low free fatty acids after 3 h (Table 1). Glucagon testing during hypoglycaemia confirmed mobilisable glycogen stores, and short ACTH stimulation test demonstrated sufficient corticotropic function. Diazoxide proved ineffective. Eight feeds during daytime and continuous overnight percutaneous feeding were required to maintain euglycemia. Several fasting tests over two years confirmed hypoketotic hypoinsulinaemic hypoglycaemia.

The abdominal mass observed at birth was surgically removed at the age of 2 months due to growth on serial imaging. Histology revealed a high-grade adenocarcinoma. Serum IGF-2 was normal. Removal of the tumour did not correct hypoglycaemia. Liver and right adrenal gland were biopsied intraoperatively and were histologically normal.

At the age of 2 years left-sided hypertrophy was observed, and MRI imaging revealed left-sided organomegaly, with no other abnormalities in the liver, pancreas, or adrenals. Cystic nephropathy was unchanged. At the age of 14 years, she had a fasting tolerance of around 5 h. Overnight she was waking once to consume fruit juice with added starch.

Individual 6 (I6) was born to non-consanguineous British parents at 36 + 1 weeks gestation. Hypoglycaemia of 2.2 mmol/L was recorded soon after birth and he was admitted to the neonatal intensive care unit where he initially required intravenous glucose. He was born with multiple jejunal atresia requiring surgery, which was complicated by small bowel perforation (Table 1). Parenteral feeding was required over the first few months due to short gut syndrome, leading to fatty liver. A diagnostic fast at the age of 6 weeks revealed hypoketotic, hypoinsulinaemic hypoglycaemia with inappropriately low free fatty acids (Table 1). At the age of 18 months, he was able to tolerate an 8 h fast allowing overnight feeding to be reduced and eventually stopped. He has right sided hemihypertrophy more noticeable in the upper than lower limbs, and is developing normally at 4.5 years’ old.

### Genetic studies

#### Individual 1

Next-generation sequencing (NGS) revealed no causal variant in genes implicated in CHI. Targeted analysis revealed a heterozygous variant in *PIK3CA* (c.323G>A p.(Arg108His)) in ~ 50% of 597 reads in leukocyte DNA, confirmed on Sanger sequencing of buccal DNA. Published functional studies have shown p.(Arg108His) to increase PI3K activity [16], permitting its classification as pathogenic using ACMG criteria.

#### Individual 2

Targeted NGS did not reveal any causative variant in genes associated with CHI. Trio exome sequencing of I2 and his unaffected parents did not identify plausible germline or mosaic *de novo*, compound heterozygous, or homozygous variants. Rare variants found are depicted in Supplementary Table 1.

#### Individual 3

NGS revealed a *de novo* variant in *PIK3CA* (c.2740G>A p.(Gly914Arg)) in 25% of 452 reads in leukocyte DNA, suggesting mosaicism [17–20]. Analysis of buccal DNA confirmed the variants showing an allele frequency of almost 50%. The variant was classified as pathogenic by ACMG criteria [21].

#### Individual 4

Constitutional pathogenic *AKT2* variants, mosaicism for the *AKT2* c.49G > A (p.Glu17Lys) “hotspot” variants, and mosaic variants in *PIK3CA* and other growth-related genes were ruled out. Exome sequencing as a trio with parents was undertaken however no rare, plausibly functional *de novo*, homozygous or compound heterozygous variants remained after filtering.

#### Individual 5

DNA methylation studies performed by MS-MLPA revealed no epigenetic or copy number abnormalities at chromosome 11p15 [22]. On Sanger sequencing, no pathogenic mutations in *AKT2*, *IGF1*, *IGF2*, *INSR*, *IGF1R*, and *IGF2R* were identified from lymphocyte DNA. High-depth NGS of genomic DNA from cultured fibroblasts, using a customised panel of cancer and overgrowth-related genes, including the whole coding sequence of *PIK3CA*, showed no pathogenic mosaic variants. Exome sequencing of the proband and unaffected parents showed five high probability *de novo* potentially function-altering coding variants with a minor allele frequency below 1 in 100,000 in controls (Supp. Table 2). Two of the genes have been implicated in autosomal recessive diseases, and heterozygosity is unlikely to be pathogenic (*PROP1, NDUFAF5*), while for the 3 remaining variants a causative role was unconvincing based on literature review, variant frequency in cancer databases, and documented associations of loci with human traits (*FRMPD2, PCDH1, RHOT1*). Three homozygous variants were also identified (Supp. Table 2). Two are in genes associated with spinocerebellar ataxia (*ATXN3, ATXN7*), while one is in *FAM3A*, encoding a cytokine-like protein that has been associated with modulation of PI3K signalling. Further individuals will be required to strengthen a role of *FAM3A* in hypoinsulinaemic hypoglycaemia.

#### Individual 6

Microarray studies revealed no chromosomal abnormalities. Beckwith-Wiedemann syndrome was excluded through sequencing and methylation studies of *CDKN1C*, *KvDMR1*, *H19DMR*, and *UDP11*, and no mutations were identified on sequencing *AKT2*. NGS of the family trio identified no high probability, *de novo*, potentially function-altering variants nor any plausibly disease-causing homozygous variants, while rare, plausibly functional compound heterozygous variants (CADD score c.21 for both variants) were found only in *TTN*, encoding the extremely large cytoskeletal Titan protein. These were not deemed a plausible cause of the syndrome based on the well understood functions and disease associations of *TTN*. Review of all rare SNVs in PI3K-AKT-mTOR pathway genes revealed heterozygous mutations in *MTOR* (c.861 C > T; p.His262Tyr; CADD score 28.9; GnomAD MAF 0) and *MLST8* (c.35 C > T; p.Pro12Leu; CADD score 23.5; GnomAD MAF 0), confirmed by Sanger sequencing, which were each shown to be inherited from a different unaffected parent (Supp. Figure 1). In the available cryo-EM structure of dimeric mTORC1 there is no obvious interaction between MLST8 and the domain of mTOR where the individual’s mTOR variant lies, arguing against direct functional interaction between the variants [23, 24].

### PI3K-AKT-mTOR pathway activity in dermal fibroblasts

Dermal fibroblasts were available for I4 and I5. These were investigated for basal hyperactivation of PI3K/AKT by phospho-ELISA, but no hyperphosphorylation of AKT nor downstream kinase GSK3β was found in either individual. Indeed, there was a significant decrease in basal phosphorylation at all three sites, possibly due to lower GSK3β expression (Supp. Figure 2).

## Discussion

Hypoglycaemia has recently been recognized as a significant manifestation of segmental overgrowth syndromes caused by pathogenic variants in the PI3K-AKT-mTOR signalling cascade [3, 4, 7, 13, 19]. It has therefore been suggested that blood glucose surveillance is incorporated into the diagnostic workup of individuals with segmental overgrowth in some circumstances [11, 13].

Here, we present six individuals with severe hypoketotic, hypoinsulinaemic hypoglycaemia. Given the rarity of this metabolic footprint, some of the individuals were initially misdiagnosed/-classified as having CHI, particularly those without syndromic features. Recognizing their distinct metabolic profile prompted us to screen for mutations activating post receptor insulin signalling, which leads to identification of pathogenic variants in two individuals. One individual (I3) had somatic features suggestive of the milder end of the *PIK3CA*-related overgrowth spectrum (PROS), and a mosaic *PIK3CA* variant was consistently detected, confirming the suspicion of MCAP syndrome [4, 13]. In contrast, none of the clinical features of I1, except for hypoinsulinaemic hypoketotic hypoglycaemia, pointed towards dysregulation of growth-related signalling pathways. However a *de novo PIK3CA* variant (c.323G>A p.(Arg108His)) with an allele frequency of 50% was found suggesting a germline variant rather than postzygotic mosaicism. The same amino acid change, p.(Arg108His), has previously been described in an endometrial tumour sample, and functional studies showed it to increase AKT phosphorylation, with corresponding effects on downstream signalling [16]. To our knowledge, this is the first individual reported with isolated hypoketotic hypoinsulinaemic hypoglycaemia due to a germline activating variant in the PI3K pathway, but without associated overgrowth.

*PIK3CA* variants in syndromic PROS individuals are almost invariably postzygotic and mosaic, and it is the patchy distribution of the mutation that drives the asymmetric overgrowth. Di Donato et al. reported an individual with a *de novo* germline *PIK3CA* variant, however, associated with macrosomia, macrocephaly, and minor brain anomalies. This individual lacked typical segmental overgrowth, vascular and digital anomalies , and no evidence of hypoglycaemia was reported [25]. In combination with our report this underlines that constitutional activating *PIK3CA* variants do exist, and in such cases cardinal clinical manifestations of PROS including asymmetric or segmental overgrowth may be absent. We suggest that a more diffuse distribution or even constitutional occurrence of *PIK3CA* variants may shift the phenotype from growth-dominated disorders to primarily metabolic disorders. Those managing neonatal and infantile hypoglycaemia should be alert to this presentation.

No genetic diagnosis has been made in several of the individuals described here, despite their distinct and often very severe metabolic phenotype, distinct from established hypoglycaemic disorders, and despite striking syndromic features in some, including cystic nephropathy and congenital adrenal carcinoma in one, and jejunal atresia in another. Failure to identify causal mutations may have several possible explanations including (a) incomplete exome coverage and/or overly stringent data filtering, (b) low-grade somatic mosaicism for the causal mutation(s) (e.g. within the liver) escaping detection in the blood/tissue samples analysed, (c) presence of non-coding causal mutations, or (d) an epigenetic disease mechanism. A hypothetical mechanism for the hypoglycaemia and jejunal atresia of I6 is suggested by mutation of conserved residues in both *MTOR* and *MLST8*. Their gene products interact as part of the mTORC complexes, with MLST8 more important for mTORC2 than mTORC1. mTORC2 acts immediately downstream from PI3K, and phosphorylates AKT at Ser 473/474 to achieve its full activation in concert with PDK1 [26–28]. However, PI3K-AKT-mTOR signalling has not been directly assessed in affected tissue, the amino acid residues affected do not interact directly with each other according to structural modelling, and a statistical genetic case cannot be made for such rare mutations. Nevertheless, given the critical role of mTORCs in the pathway that causes other forms of hypoketotic hypoglycaemia, the digenic variants remain a conceivable cause of the observed syndrome. This requires further study.

Our findings suggest that the disease spectrum of hypoketotic hypoinsulinaemic hypoglycaemia may be wider still than currently realised. Ascertainment may be reduced by the striking clinical overlap with “classical” CHI, which sometimes leads to misclassification. Concerted genetic and tissue signalling studies of further affected individuals are required to delineate the molecular pathomechanism in unexplained cases. Current aetiological uncertainty in some of the cases we describe is also seen in CHI, which remains a genetically unsolved clinical/metabolic entity in a significant proportion of individuals until today [29, 30].

## Conclusions

In conclusion, our findings expand the spectra of PI3K-related growth disorders and of congenital hypoglycaemic disorders, and emphasise that *PIK3CA* variants should be considered in individuals presenting with “CHI”-like disease but low or absent plasma insulin concentrations, even without features of overgrowth. We propose that variants in the post insulin receptor signalling cascade should be considered in the differential diagnosis of congenital hypoglycaemic disorders even if typical features suggestive of mosaic overgrowth syndromes are absent. To facilitate adoption and awareness of this specific metabolic entity, we propose the term “pseudohyperinsulinism”.

### Electronic supplementary material

Supplementary Material 1.

## Data Availability

The next generation sequencing data that support the findings of this study are not openly available for reasons of patient confidentiality, based on the informed consent obtained. They are available from the corresponding author upon reasonable request, however, and are located in controlled access data storage at either Medizinische Genetik Mainz or the European Genome-Phenome Archive.
