# Supplementary material for: An expanded clinical spectrum of hypoinsulinaemic hypoketotic hypoglycaemia
Source: Orphanet J Rare Dis. 2023 Nov 16;18:360. doi: 10.1186/s13023-023-02954-5 (PMC10652530; doi:10.1186/s13023-023-02954-5)
Supplement: Supplementary file 1 — Supplementary Material 1. [file 13023_2023_2954_MOESM1_ESM.pdf]

An expanded clinical spectrum of hypoinsulinaemic hypoketotic hypoglycaemia
A Welters, SM Leiter *et al*
DOI: 10.1186/s13023-023-02954-5

Supplementary Data

Supplementary Table 1: rare variants identified in individual 2

**Rare heterozygous *de novo* variants**					
Gene	Accession No.	Nucleotide change	Protein change	Function or Disease Association	GnomAD MAF
No suggestive variants detected					
**Homozygous variants**					
Gene	Transcript No.	Nucleotide change	Protein change	Function or Disease Association	GnomAD MAF
PYGO2	NM_138300.3	c.423G>A	p.(Met141Ile)	WNT signal transduction, B-cell malignancies	1.57e-3
ZNF700	NM_001271848.1	c.212_213delGTinsAA	p.(Ser71Lys)	Zinc finger protein, unclear function	-
SAE1	NM_005500.2	c.683A>G	p.(Lys228Arg)	SUMO-activating enzyme	6.39e-4
**Compound heterozygous variants**					
Gene	Transcript No	Nucleotide change	Protein change	Function or Disease Association	GnomAD MAF
GOLGB1	NM_001256486.1	c.8582G>A/ c.3809A>C	p.(Arg2861Gln)/p.(Glu1270Ala)	Protein glycosylation, LOF mouse: cleft palate	4.3e-6/ 5.66e-5
MUC20	NM_001282506.1	c.331G>A/ c.511G>A	p.(Glu111Lys)/p.(Gly171Ser)	Kidney function, upregulated in IgA nephropathy	9.20e-4/ 2.65e-5
AHNAK	NM_001620.2	c.7439G>C/ c.476C>T	p.(Gly2480Ala)/ p.(Ala159Val)	Calcium signalling; LOF mouse: no obvious phenotype	6.72e-5/ 0
MUC16	NM_024690.2	c.38773G>A/ c.30698C>A/ c.26348C>G	p.(Ala12925Thr)/ p.(Ala10233Asp)/ p.(Ala8783Gly)	Ovarian cancer tumour antigen	5.20e-4/ 1.79e-5/ 2.71e-4
HAPLN4	NM_024690.2	c.418C>T/ c.80C>A	p.(Arg140Cys)/ p.(Ala27Glu)	Hyaluronan proteoglycan link protein; tricuspid valve prolaps	7.78e-5/ 3.18e-4
**x-chromosomal hemizygous in index individual, heterozygous in mother**					
Gene	Transcript No	Nucleotide change	Protein Change	Function or Disease Association	GnomAD MAF
LHFPL1	NM_178175.3	c.482-7C>T	p.?	Unclear function	6.72e-4
RBMXL3	NM_001145346.1	c.1963G>A	p.(Asp655Asn)	RNA binding motif protein	7.45e-4
SRPK3	NM_014370.3	c.907G>A	p.(Asp303Asn)	Serine/arginine specific protein kinase	6.2e-6
CMC4	NM_001018024.2	c.205T>C	p.(*69Glnext*13)	Cx9C motif-containing protein 4	3.72e-4

DOI: 10.1186/s13023-023-02954-5

Supplementary Table 2: rare *de novo*, compound heterozygous and homozygous genetic variants identified in individual 5

Gene	Accession No.	Nucleotide change	Protein Change	CADD score	Function or Disease Association	GnomAD MAF
**Rare *De Novo* Variants**						
*PROP1**	NM_006261.5	c.604G>A	p.Arg99Ter	36	Combined pituitary deficiency (AR)	7.08e-6
*NDUFAF5**	NM_024120.5	g.13782331T>C	Splice donor	33	Mitochondrial complex 1 deficiency (AR)	0
*FRMPD2**	NM_001018071.4	c.1658C>T	p.Arg452Glu	32	Involved in epithelial polarity	0
*PCDH1**	NM_032420.5	c.1209C>T	p.Arg354His	27	Cell adhesion	3.98e-6
*RHOT1**	NM_001033566.3	c.1513G>A	p.Cys429Tyr	23	Involved in mitochondrial motility; Possible Parkinson's Disease association	0
**Rare Homozygous Variants**						
Gene	Accession No.	Nucleotide change	Protein change	CADD score	Function or Disease Association	
FAM3A	NM_021806.4	c.622G>A	Pro52Leu	23	Cytokine-like protein; implicated in PI3K signalling	7.18e-6
ATXN3	NM_004993.6	c.916insG	Leu307fs	22	Machado Joseph Disease (AD; CAG repeats)	0
ATXN7	NM_001377405.1	c.657A>C	Gln35Pro	23	Spinocerebellar ataxia (AD; CAG repeats)	0

*confirmed to be *de novo* by Sanger sequencing of family trio

Supplementary Figure 1: rare MLST8 and MTOR variants in individual 6 and parents

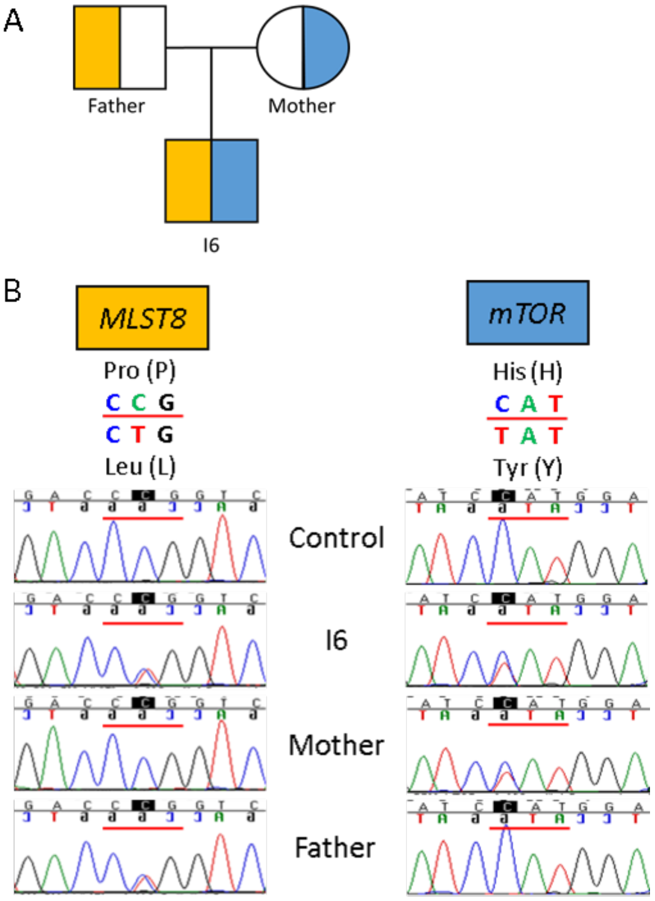

C

Gene	Accession No.	Nucleotide change	Protein change	CADD score	Comments	GnomAD MAF
MTOR	NM_004958.4	c.861G>A	His262Cys	26.7	Smith-Kingsmore syndrome, brain development, somatic cancer mutations	0
MLST8	NM_022372.6	c.389C>T	Pro12Leu	23.3	Tuberous sclerosis 1, subependymal glioma	2.52e-5

A) Inheritance pattern of MLST8 and mTOR in individual 6 with **B)** confirmatory Sanger sequencing and **C)** details of detected variants.

DOI: 10.1186/s13023-023-02954-5

Supplementary Figure 2: no baseline AKT or GSK3β hyperphosphorylation in dermal fibroblasts from individuals 4 and 5

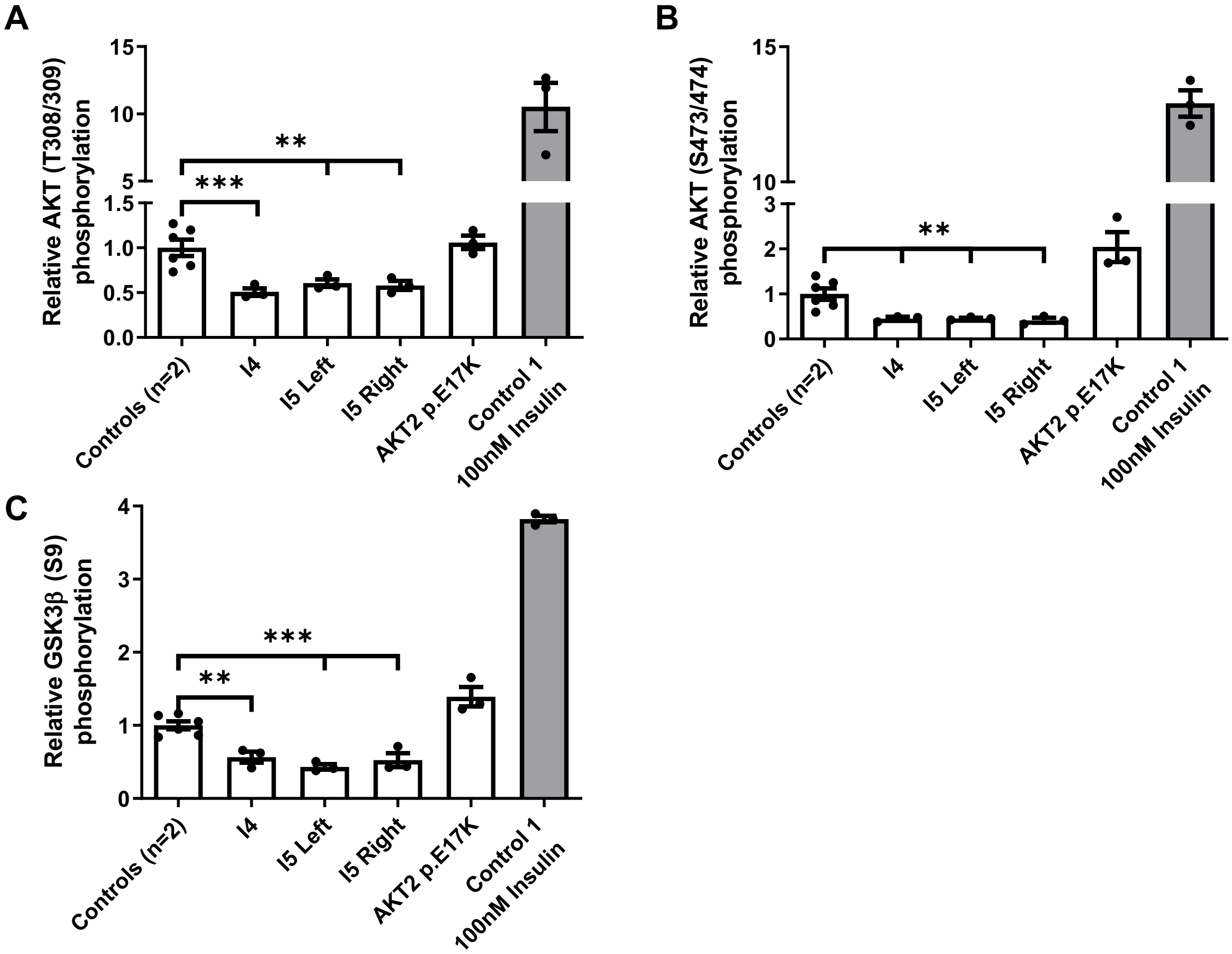

A-C) Following 24 hours of serum starvation phosphorylation was assessed of AKT at Thr308/309 **(A)**, Ser473/474 **(B)**, and GSK3β at Ser9 **(C)** in dermal fibroblasts from individuals 4 (I4) and 5 (I5) using ELISA of cell lysates. Data represents pooled and normalised results from three independent experiments plotted as mean ± SEM. Phosphorylation was compared using a one-way ANOVA followed by post hoc Dunnett's test to compare the patient cell lines to two pooled controls. Insulin stimulation is shown for illustration only and was excluded from this statistical analysis.
**: $p<0.01$, ***: $p<0.001$
